# Implementing the World Health Organization Pandemic Influenza Severity Assessment framework—Singapore's experience

**DOI:** 10.1111/irv.12680

**Published:** 2019-10-17

**Authors:** Rachael Pung, Vernon Jian Ming Lee

**Affiliations:** ^1^ Communicable Diseases Division Ministry of Health Singapore Singapore

**Keywords:** epidemic, influenza, surveillance

## Abstract

**Background:**

We report our experience in evaluating the severity of local influenza epidemics using the World Health Organization Pandemic Influenza Severity Assessment framework.

**Methods:**

We assessed the severity of influenza by monitoring indicators of influenza transmissibility, seriousness of disease and impact on healthcare resource utilisation. Indicators were described by various parameters collected weekly from eight government hospitals, 20 government and 30 private primary care clinics, and the national public health laboratory. Transmissibility and seriousness of disease indicators were each represented by multiple parameters, and alert thresholds were set at the 70th and 90th percentile of a parameter's past 2‐year surveillance data. We derived a collective measure for each indicator using the average percentile rank of the related parameters. Alert thresholds for the single impact parameter were set at predefined values and evaluated for its sensitivity, specificity and positive predictive value.

**Results:**

For the transmissibility and seriousness of disease parameters, calculation of the percentile rank was simple and independent of a parameter's underlying distribution. For the impact parameter, predefined alert thresholds had high sensitivity and specificity (>80%) but low positive predictive value (15%‐30%). Assessment scales were used to qualitatively classify the activity of an indicator as low, moderate or high together with a confidence level.

**Conclusion:**

We applied different methods for threshold setting depending on the attributes of each parameter and indicator. For indicators represented by multiple parameters, an aggregated assessment of the indicator's level of activity and confidence level of the assessment was needed for effective reporting.

## 1 BACKGROUND

Early severity assessment of pandemic influenza is helpful for guiding pandemic response actions. However, during the 2009 H1N1 pandemic, severity assessment was not standardised across countries, making it difficult to evaluate the local or global situation as the pandemic evolved.^1^ The lack of a consistent measure of severity also posed a challenge to calibrate pandemic response, which is dependent on geographical spread, clinical severity and public interest, among other factors.^1^

Through the lessons learnt from the 2009 H1N1 pandemic, the World Health Organization (WHO) has developed a framework for pandemic influenza severity assessment (PISA).^2^ PISA is a structured way of tracking influenza epidemics or pandemics. The three recommended *indicators* for monitoring severity were the transmissibility of the influenza virus, the seriousness of the disease and the impact of influenza on healthcare resource utilisation (referred to as transmissibility, seriousness of disease and impact, in the subsequent sections). By assessing severity from multiple dimensions, this encourages countries to establish surveillance at different levels of the healthcare system to create a holistic picture of an influenza epidemic or pandemic.

Using virological and surveillance data from different sources, the severity of each indicator can be represented by more than one type of data, or *parameter*. The choice of parameters may vary across countries due to different data availability, of which some require substantial resource to collect. While the challenge of data comparison remains, PISA plays an essential role—to promote enhanced surveillance and increase information sharing among public health officials during an influenza epidemic or pandemic.

### 1.1 Influenza surveillance in Singapore

Singapore, a city‐state in South East Asia, is a major global travel hub with over 18 million tourist arrivals^3^ and a population of over 5.6 million in 2018.^4^ It has a high population density of over 8000 people per square kilometre, which may facilitate the spread of contact transmissible and airborne diseases such as influenza.

Locally, influenza A (H1N1) pdm09, A (H3N2) and influenza B viruses circulate year‐round. Following the 2009 influenza pandemic, we expanded our influenza surveillance network and encouraged government and private primary care clinics to participate in the National Influenza Surveillance Programme. To date, 20 government primary care clinics, providing about 20% of primary healthcare services in the population,^5^ and 30 sentinel clinics spread across the country out of 1400 private primary care clinics are enrolled in the programme. Our influenza surveillance network also comprises eight acute government hospitals, providing about 80% of all acute care hospital services in the population^5^ and the National Public Health Laboratory (NPHL).

In this paper, we document Singapore's experience in developing and evaluating the PISA indicators and parameters, and this would provide other countries with suggestions that they can use in developing their own indicators.

## 2 METHODOLOGY

### 2.1 Data sources

A wide range of parameters were reported weekly to the Ministry of Health (MOH) and considered for PISA (Table 1). Influenza transmission in the community was monitored using the average daily attendance for acute respiratory infection (ARI) and the average daily attendance for influenza‐like illness (ILI) at the government primary care clinics. An ARI diagnosis was made when a case had at least one acute respiratory symptom such as cough, sore throat and coryza, while an ILI diagnosis was made when a case had a fever of ≥38.0°C and cough, with onset within the last 10 days. The average daily attendance for ARI and average daily attendance for ILI at the government primary care clinics were used, instead of the weekly attendances, to offset the effect of public holidays and clinic closure on weekends.

**Table 1:** Parameters considered for assessing severity of influenza

Indicator	Singapore parameters considered	Data source	WHO recommended parameters
Transmissibility *How many people in a population get sick from influenza on a weekly basis*	Average daily attendance for ARI	20 government primary care clinics	Weekly ILI or MAARI cases as a proportion of total visits or incidence rates. Weekly percentage of respiratory pathogen samples testing positive for influenza. Composite (product) of weekly ILI or MAARI and weekly percentage positivity rates for influenza
	Average daily attendance for ILI		
	Proportion of respiratory samples positive for influenza over a 4‐weekly moving interval	20 government and 30 private primary care clinics	
	Estimated average daily number of influenza‐positive ILI cases		
Seriousness of disease *How severely sick an individual gets when infected with the influenza virus*	Weekly number of ARI ED attendances	8 government acute hospitals	SARI/ARI or ILI ratio Cumulative death: hospitalisation ratio (ideally for confirmed influenza) Cumulative ICU: hospitalisation ratio (ideally for confirmed influenza)
	Weekly number of ARI ED admissions		
	Weekly proportion of ARI ED attendances resulting in admission		
	Weekly number of pneumonia ED attendances		
	Weekly number of pneumonia ED admissions		
	Weekly proportion of pneumonia ED attendances resulting in admission		
Impact *How the influenza epidemic or pandemic affects the healthcare system (and society)*	Weekly number of laboratory‐confirmed influenza cases admitted to ICU or died	8 government acute hospitals	Weekly or monthly number or proportion of SARI cases with percentage flu‐positive among SARI cases Weekly excess pneumonia & influenza (P&I) or all‐cause mortality stratified by age. Weekly number of confirmed influenza cases admitted to ICU, or weekly number of confirmed influenza cases admitted to hospital.

Abbreviations: ARI: acute respiratory infection; ED: emergency department of a government hospital; ICU: intensive care unit of a government hospital; ILI: influenza‐like illness; MAARI: medically attended acute respiratory illness; SARI: severe acute respiratory infection.

Consent was sought for the collection of respiratory samples from all patients if they received outpatient consultation at a government or private primary care clinics that are enrolled in the National Influenza Surveillance Programme and presented with ILI. These samples were routinely submitted to the NPHL and tested using the FilmArray Respiratory Panel and/or real‐time reverse transcription‐polymerase chain reaction (RT‐PCR) to detect respiratory viruses. The weekly number of samples was small as not all identified patients participated in the surveillance, and hence, we pooled the results across four weeks and monitored the proportion of respiratory samples positive for influenza over a 4‐weekly moving interval.

As not all ILI attendances at the government primary care clinics were attributed to influenza, we explored using the product of the average daily attendance for ILI and weekly proportion of respiratory samples positive for influenza to estimate the average daily number of influenza‐positive ILI cases at the government primary care clinics. We also collect parameters from the eight acute government hospitals comprising of the weekly number of ARI Emergency Department (ED) attendances and admissions, the weekly number of pneumonia ED attendances and admissions, and the weekly number of laboratory‐confirmed influenza cases admitted to the intensive care unit (ICU) or died. The former two parameters were collected through MOH’s healthcare utilisation database while the latter was compiled by a team of healthcare professionals in each hospital and forwarded to MOH.

A time series plot of each parameter was used to illustrate the parameter's variability during each seasonal epidemic and surveillance artefacts arising from reporting changes. These two factors were considered in the final selection of parameters used for PISA reporting.

### 2.2 Assessing the transmissibility and seriousness of disease indicators’ level of activity

As the transmissibility and seriousness of disease indicators were represented by more than one parameter, an overall measure of each indicator's level of activity and the confidence of the indicator was necessary for weekly reporting.

For a parameter, we calculated the *percentile rank* or the *percentile* of an observed value with respect to the previous 2‐year historical data (eg the percentage of data from January 2016 to December 2017 that were equal or lower than a weekly parameter data collected in 2018). We limited the comparison to 2‐year historical data due to recent changes in data extraction methods. Let *x_w_* denote the observed value of a parameter and *p_w_* denote the percentile of that observed value in week *w* of a year. Also, let *h* denote the historical data in the previous 2 years.

$$p_{w}\left\{\begin{array}{c} =\;0\;if\;x_{w}\;<\min\;\left(h\right)\\ =\frac{\text{CF}+0.5f}{n}\;\\ \;\;=100\;if\;x_{w}\;>\max\;\left(h\right) \end{array}\right.$$

where CF is the number of values in h that is below $x_{w}$ (ie cumulative frequency). f is the number of values in *h* that is equal to *x_w_* (ie frequency). *n* is the number of values in *h*.

To quantify an indicator's level of activity, we calculated the average percentile of all the parameters of an indicator. On a scale from zero to 100, percentile values of 70 and 90 were used as cut‐offs (ie alert thresholds) to provide three classifications of an indicator's level of activity depending on where the average percentile value lies on the scale (low: [0, 70]; moderate: [70, 90]; high: [90, 100]). Furthermore, the distance of the average percentile value from the cut‐offs percentiles provided a measure of confidence—the further away, the average percentile is from an alert threshold, the greater the confidence in the assessment of an indicator's level of activity and vice versa.

### 2.3 Assessing the impact indicator's level of activity

The weekly number of laboratory‐confirmed influenza cases who were admitted to the intensive care unit (ICU) or died is the only impact parameter, and we used data from January 2011 to December 2017 for threshold setting due to the absence of reporting artefacts over the years. The discrete data had a small range of observed values, and hence, we used a different approach to set the alert thresholds and to ensure that alert thresholds had integer values. A sustained high (moderate) influenza activity is said to occur when the impact parameter values remain above the high (moderate) alert thresholds for 2 weeks after the first alert week. We set alert thresholds at predefined values and tested two different scenarios. In the first scenario, the moderate and high alert thresholds were set at three and six, respectively. In the second scenario, they were revised to four and six, respectively. We evaluated key performance metrics of sensitivity, specificity and positive predictive value (PPV) of a threshold to assess the threshold's ability to provide early warning prior to the peak of an influenza season.^6^ The sensitivity was the proportion of sustained high influenza activity with a moderate alert raised in at least one of the 2 weeks prior to crossing the high alert threshold. The specificity was the proportion of weeks with no alerts during the baseline influenza periods. The PPV for high (moderate) influenza activity was the proportion of true high (moderate) alerts among all high (moderate) alerts.

## 3 RESULTS

### 3.1 Parameters selected for PISA reporting

**Figure 1:**
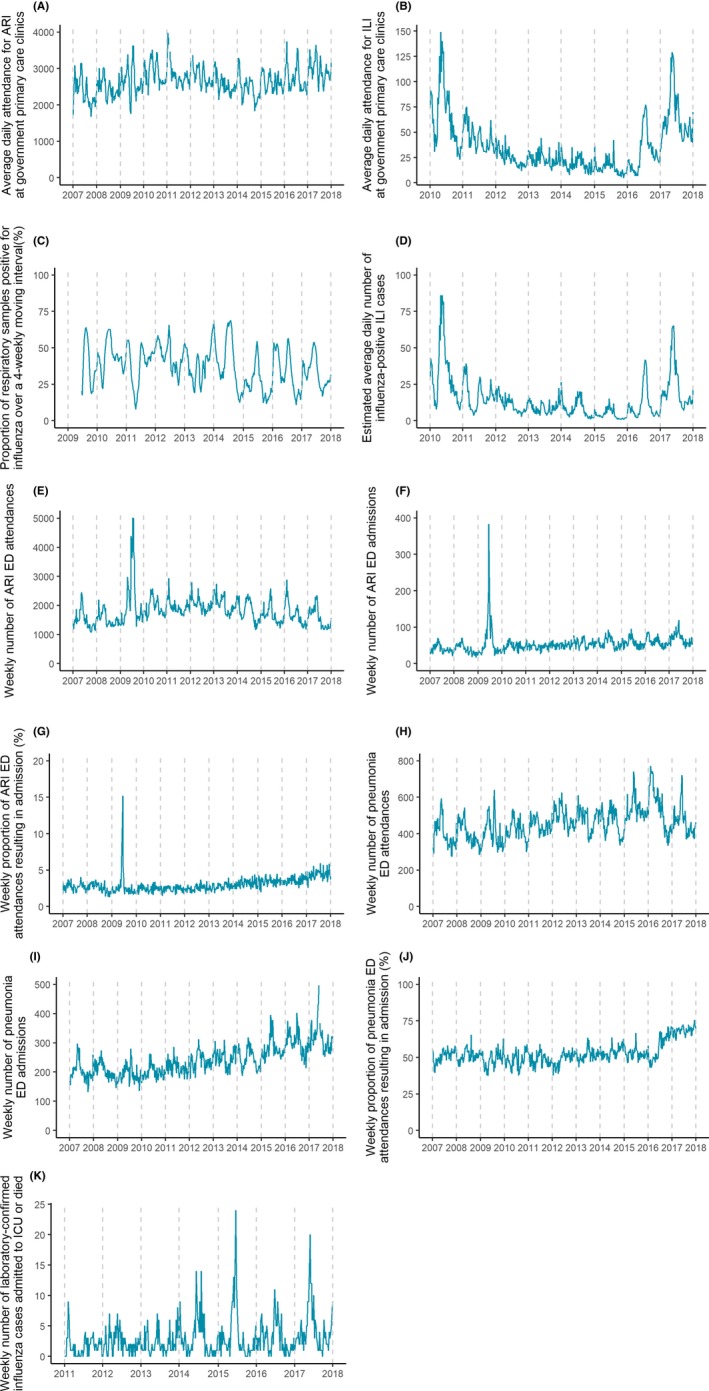
Time series plots of (A) average daily attendance for ARI at government primary care clinics, (B) average daily attendance for ILI at government primary care clinics, (C) proportion of respiratory samples positive for influenza over a 4‐weekly moving interval, (D) estimated average daily number of influenza‐positive ILI cases, (E) weekly number of ARI ED attendances, (F) weekly number of ARI ED admissions, (G) weekly proportion of ARI ED attendances resulting in admission, (H) weekly number of pneumonia ED attendances. cont'd (I) weekly number of pneumonia ED admissions, (J) weekly proportion of pneumonia attendances at the ED resulting in admission, (K) weekly number of laboratory‐confirmed influenza cases admitted to ICU or died

Time series plots of the parameters in Table 1 are shown in Figure 1. The average daily attendance for ARI at the government primary care clinics (Figure 1A) exhibits a multimodal distribution as it is influenced by the activity of other respiratory viruses, and the seasonal peaks of these viruses might not be in sync with the influenza seasons. While the average daily attendance for ARI at government primary care clinics is less representative of the local influenza transmissibility as compared to its ILI counterpart, it is still important to track it as influenza with low clinical severity may appear more frequently as ARI.

From 2011 to 2015, the average daily attendance for ILI at the government primary care clinics declined (Figure 1B) and this could be attributed to gradual underreporting after the 2009 H1N1 pandemic. In 2016, ILI case definition was reiterated to all government primary care clinics leading to a rise in the measure. The estimated average daily number of influenza‐positive ILI cases at the government primary care clinics was not chosen for as a parameter for transmissibility eventually as it is a repeated representation of its individual components and any variations caused by reporting artefacts will affect its interpretation. Additionally, the proportion of total visits attributed to ARI or ILI, as recommended by WHO, was not monitored as it represented the burden of influenza compared to other diseases instead of the transmissibility of the virus.

The proportion of respiratory samples positive for influenza over a 4‐weekly moving interval was the only laboratory‐confirmed influenza parameter for the transmissibility indicator. Higher local influenza activity was observed from May to July and from November to January, and generally coincides with the winter in the Southern and Northern Hemisphere, respectively (Figure 1C). Seasonal fluctuations were observed in the weekly attendances and admissions at the emergency department (ED) of acute government hospitals for both ARI and pneumonia (Figure 1E‐J). During the 2009 influenza pandemic, the weekly number of ARI ED attendances (Figure 1E) and admissions (Figure 1F) and, consequently, the weekly proportion of ARI ED attendances resulting in admission (Figure 1G) indicated a clear spike. One‐off adjustment in 2016 was also observed in the pneumonia parameters (Figure 1H‐J) due to change in the disease classification and coding systems of some hospitals. The weekly proportion of ARI or pneumonia ED attendances resulting in admission (Figure 1G,J) was selected as parameters for the seriousness of disease as it indicated the extent to which individual gets sick and required hospital care.

The weekly number of laboratory‐confirmed influenza cases who were admitted to the ICU or died described the impact of influenza on healthcare resource utilisation and was the only parameter for the impact indicator. Influenza mortality was a component of the parameter as the management of critically ill patients in general wards could also be resource intensive (eg manpower needed for frequent monitoring a patient's progress and calibration of treatment). Sharp peaks in this parameter were typically observed during May to July, coinciding with winter in the Southern Hemisphere (Figure 1K).

### 3.2 Performance of the impact parameter alert thresholds

The weekly number of laboratory‐confirmed influenza cases that were admitted to ICU or died ranged from 0 to 24 (Figure 1K). When the moderate and high alert thresholds were predefined at an integer value of 3 and 6, respectively, 29 moderate alerts and 19 high alerts were raised from 2011 to 2017. Of these alerts, nine moderate alerts and four high alerts preceded sustained moderate or high influenza activity (ie 31.0% and 21.1% PPV for moderate and high alert threshold, respectively). In all four of the observed sustained high influenza activity, a moderate alert was made known at least one week prior to trigger of the high alert (ie 100% sensitivity). No alerts were made in 83 of the 103 weeks of baseline influenza activity (ie specificity of 80.6%).

In the second scenario, the moderate alert threshold was increased to a value of 4, and 20 moderate alerts were raised from 2011 to 2017. Of these alerts, three resulted in sustained moderate influenza activity (ie PPV of moderate alert threshold of 15.0%). The sensitivity remained at 100%. No alerts were made in 138 of the 156 weeks of baseline influenza activity (ie specificity of 88.5%).

### 3.3 Assessment scale for indicators

**Figure 2:**
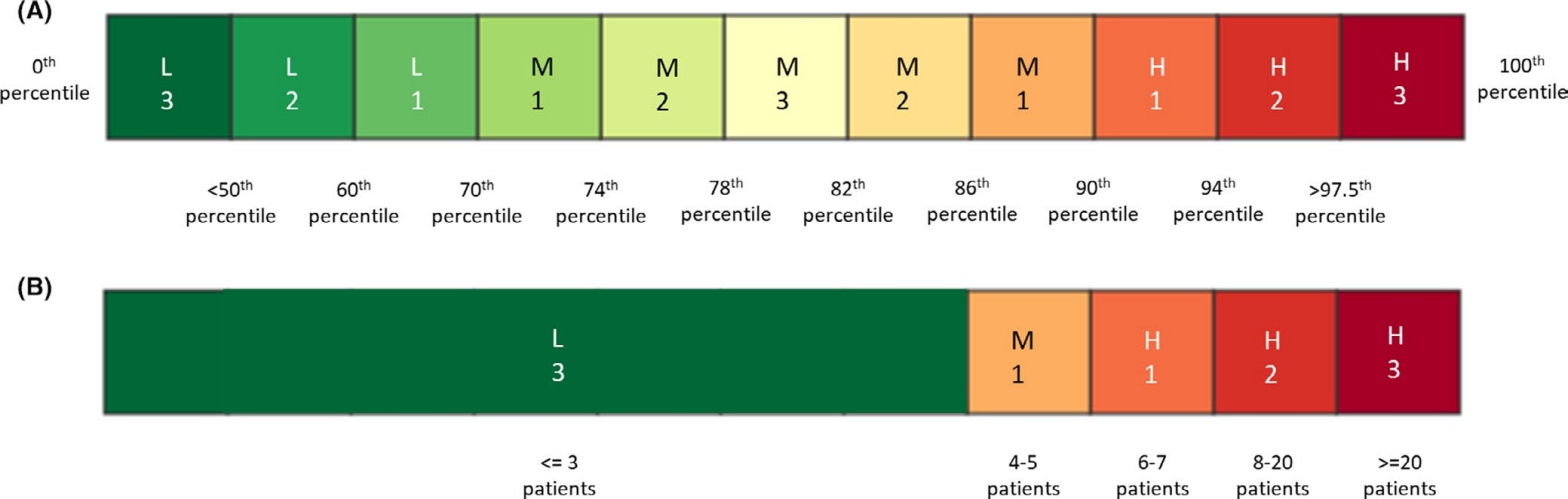
Assessment scale for (A) transmissibility and seriousness of disease indicators, (B) impact indicator. Severity of an indicator is classified as L: low, M: moderate, H: high. Confidence level of an indicator is classified as 1: low, 2: medium, 3: high

Figure 2A shows the assessment scale used to qualitatively classify the level of activity of the transmissibility and seriousness of disease indicators*.* The coloured scale showed gradual transition from dark green to dark red signifying increasing levels of activity of an indicator. The small range of discrete values observed in the single impact parameter limits our ability to provide multiple, meaningful cut‐offs, and hence, a separate assessment scale was created (Figure 2B) based on the results in the previous section, *Performance Matrices for the Impact Parameter Alert Threshold.* Table 2 illustrates the weekly PISA results from E‐week 1 to 10 of 2018.

**Table 2:** Weekly PISA results

PISA reporting:		Transmissibility			Seriousness of disease			Impact		
Year	E‐week	Risk	Confidence		Risk	Confidence		Risk	Confidence	
2018	1	Moderate	Low		Low	Medium		High	Medium		
2018	2	Moderate	Low		Low	Medium		High	Low		
2018	3	High	Low		Moderate	High		Moderate	Low		
2018	4	High	Low		Low	High		Low	High		
2018	5	High	Low		Low	High		Low	High		
2018	6	High	Low		Low	High		High	Low		
2018	7	Moderate	Low		Low	Low		Low	High		
2018	8	Moderate	High		Moderate	Low		Moderate	Low		
2018	9	Moderate	Low		Low	Low		Low	High		
2018	10	Low	Low		Moderate	Medium		Moderate	Low		

## 4 DISCUSSION

Influenza surveillance in Singapore spans all acute government hospitals, all government and some private primary care clinics. PISA indicators representing the transmissibility of influenza virus, seriousness of disease or the impact of influenza on healthcare resource utilisation highlight different aspects of influenza activity. This provides comprehensive surveillance of the severity of a current influenza season and allows the ministry to determine the extent of public health responses required to manage the transmission and to protect vulnerable populations.

Of the three indicators, transmissibility has the widest variety of parameters customised for each country's setting. In Singapore, data on the ARI and ILI attendance at government primary care clinics are conveniently extracted from various healthcare surveillance platforms for weekly reporting. In other countries, the number of callers to public health hotline reporting ILI^7^, ^8^ or prescription records^9^, ^10^ were also explored as means to characterise the extend of spread when complemented with data sources from healthcare institutions.

Key challenges remain in achieving a representative indicator for seriousness of disease in Singapore. The weekly proportion of ARI or pneumonia ED attendances that were hospitalised were chosen to illustrate the severity of each condition, but the absence of hospital laboratory surveillance data limits our ability to verify the infection status of each patient. Spikes in the weekly proportion of ARI ED attendances that were hospitalised (Figure 1G) could be attributed to changes in health‐seeking behaviour, reporting habits of physicians and higher tendency to admit a patient during a pandemic, though extent of influence has yet to be studied.

The cumulative number of patients tested positive for influenza admitted to ICU is a component to some WHO recommended parameters in Table 1. This component is limited by the number of ICU beds, and ICU admission of a severe influenza case is subjected to competing requirements of other non‐influenza‐positive patients depending on severity. Furthermore, depending on a hospital's technological and manpower capability, critical care could be provided in general wards. An improved measure would be the ratio of cases fulfilling the definitions of complicated or severe influenza^11^ to the number of influenza‐positive admission. However, the feasibility of measuring this is dependent on the healthcare system's ability to integrate laboratory and epidemiological data.

Severity assessment has been largely focused on developing different methods to establish alert thresholds that signal the start or the end of an influenza season. Based on the characteristics of a parameter,^12^ a variety of methods such as the Moving Epidemic Method (MEM) or cumulative sum control charts (CUSUM) have been developed for early epidemic detection. For Singapore, thresholds setting methods were chosen based on the data characteristics. Regular review and enhancement of data extraction methods helps to improve accuracy of the parameters but inevitably creates artefacts in the historical surveillance data and limits the feasibility of using methods that require long history of surveillance data. Hence, for transmissibility and seriousness of disease parameters, the moderate and high alert thresholds of a year were set using the 70th and 90th percentiles of the past 2‐year data. For the impact parameter, the moderate and high alert thresholds were set using predefined integer values. The PPV of the thresholds was poor and implied that in many occasions, there was no sustained moderate or high influenza activity occurring after a moderate or high alert was triggered. The moderate threshold was eventually set at four as about 70% of the historical data was below this value, and a moderate alert was triggered before the onset of all sustain high influenza activity.

In this paper, we also presented an assessment scale, which provides a combined measure of an indicator's level of activity and the confidence level of the assessment. With more than one parameter serving as proxies for an indicator, the method of providing an aggregated assessment for an indicator remains undocumented in PISA. Furthermore, the confidence of an indicator's assessment is part of PISA reporting, but its interpretation is multifaceted. It is dependent on, but not limited to, reporting biases, timeliness and agreement between the parameters. The first two factors are related to the reliability of the information provided at various sentinel sites and can be improved with a structured data collection process. On the contrary, the agreement between the parameters is intrinsic to the influenza activity of a season. Each parameter is a unique proxy of an indicator and might be influenced by the activity of other respiratory viruses. Thus, a high agreement between the parameters provides greater certainty to the measure of an indicator's level of activity.

The quantification of an indicator's level of activity is achieved by averaging the percentile rank of all the parameters representing an indicator with the assumption that all parameters were equally informative. However, ARI parameters can be influenced by the activity of other respiratory viruses. As such, there may be occasions where the average daily attendance for ARI was high but the same was not observed for ILI surveillance data. However, it is still important to track the ARI attendances at the government acute hospitals and primary care clinics as it potentially informs us of any changes in the clinical representation of influenza cases. One possible way of overcoming this challenge is to assign weights to each parameter based on its importance in assessing the local influenza situation. The weighted average percentile rank could be computed to represent an indicator's level of activity.

In addition, when a parameter is higher (or lower) than the historical maximum (or minimum), the percentile of that parameter's data was capped at 100 (or zero). Taking the average percentile values of all parameters of an indicator then helps to ensure that the extreme results of one parameter would not dominate the measure of an indicator but allows it to skew the measure towards a higher (or lower) classification of the indicator's level of activity.

The confidence assessment did not consider the number of parameters used to represent an indicator. It is possible for an indicator's parameter to reflect a very different level of activity compared to the rest of the parameters. In situations where there are few parameters representing an indicator, the extreme parameter is likely to skew an indicator's level of activity towards an extreme. The effect of the extreme parameter on the indicator's level of activity would attenuate when the number of parameters representing an indicator increases. Also, any sustained occurrence of abnormalities needs to be highlighted and the interpretation of the average percentile under such conditions should be done with caution.

## 5 CONCLUSION

We share Singapore's practices in the weekly assessment of PISA indicators. For indicators represented by multiple parameters, a collective assessment of the indicator's level of activity and the confidence level of this assessment were necessary. Here, we have introduced an assessment scale to accomplish both objectives. We placed priority in creating a simple collective assessment for a complex indicator. The choice of parameters, sampling criteria and case definitions were regularly reviewed and updated to ensure consistent performance of our surveillance system. Our method of PISA reporting could be applied in other countries, with parameters chosen based on the resources of the country, and the assessment scale customised to the local setting.

## References

[1] World Health Organization . Strengthening response to pandemics and other public‐health emergencies: report of the Review Committee on the Functioning of the International Health Regulations (2005) and on Pandemic Influenza (H1N1) 2009 https://apps.who.int/iris/bitstream/handle/10665/75235/9789241564335_eng.pdf;jsessionxml:id=6C57B890718C3C665CC1B36C70BC3A15?sequence=1 Accessed 15 July, 2019.

[2] World Health Organization . Pandemic Influenza Severity Assessment (PISA): a WHO guide to assess the severity of influenza epidemics and pandemics. https://apps.who.int/iris/bitstream/handle/10665/259392/WHO-WHE-IHM-GIP-2017.2-eng.pdf?sequence=1 Accessed January 5, 2019.

[3] Singapore Tourism Board . International visitor arrival statistics. https://www.stb.gov.sg/content/dam/stb/documents/statistics-marketing-insights/international-visitor-arrivals/pdf/visitor-arrivals-2018.pdf Accessed February 20, 2019.

[4] Singapore Department of Statistics . Population and population structure. https://www.singstat.gov.sg/find-data/search-by-theme/population/population-and-population-structure/latest-data Accessed February 20, 2019.

[5] Lee CE , Satkunanantham K . Singapore’s Health Care System: What 50 Years Have Achieved. Singapore: World Scientific Publishing Co., Pte. Ltd.; 2016:18.

[6] Hashimoto S , Murakami Y , Taniguchi K , Nagai M . Detection of epidemics in their early stage through infectious disease surveillance. Int J Epidemiol. 2000;29(5):905‐910. 11034976 10.1093/ije/29.5.905

[7] Vette K , Bareja C , Clark R , Lal A . Establishing thresholds and parameters for pandemic influenza severity assessment, Australia. Bull World Health Organ. 2018;96(8):558‐567. 30104796 10.2471/BLT.18.211508 PMC6083389

[8] Public Health England . Surveillance of influenza and other respiratory viruses in the UK: winter 2017 to 2018. https://assets.publishing.service.gov.uk/government/uploads/system/uploads/attachment_data/file/740606/Surveillance_of_influenza_and_other_respiratory_viruses_in_the_UK_2017_to_2018.pdf Accessed January 5, 2019.

[9] Sugawara T , Ohkusa Y , Ibuka Y , Kawanohara H , Taniguchi K , Okabe N . Real‐time prescription surveillance and its application to monitoring seasonal influenza activity in, Japan. J Med Internet Res. 2012;14(1):e14. 22249906 10.2196/jmir.1881 PMC3846340

[10] Chen JH , Schmit K , Chang H , Herlihy E , Miller J , Smith P . Use of medicaid prescription data for syndromic surveillance –‐ New York. MMWR Morb Mortal Wkly Rep. 2005;54(Suppl):31‐34. 16177690

[11] World Health Organization . WHO guidelines for pharmacological management of pandemic influenza A(H1N1) 2009 and other influenza viruses. https://www.who.int/csr/resources/publications/swineflu/h1n1_guidelines_pharmaceutical_mngt.pdf Accessed January 5, 2019. 23741777

[12] World Health Organization . Global epidemiological surveillance standards for influenza. https://www.who.int/influenza/resources/documents/WHO_Epidemiological_Influenza_Surveillance_Standards_2014.pdf Accessed January 5, 2019.

